# Relative humidity, precipitation, and outpatient visits for clinically diagnosed fungal otitis externa: evidence from a single-center time-series study in Wuxi, eastern China

**DOI:** 10.3389/fpubh.2026.1859702

**Published:** 2026-06-19

**Authors:** Xiangming Meng, Mingjing Cai

**Affiliations:** Department of Otolaryngology, Affiliated Huishan Hospital of Xinglin College, Nantong University, Wuxi Huishan District People's Hospital, Wuxi, China

**Keywords:** distributed lag non-linear model, fungal otitis externa, otomycosis, outpatient visits, precipitation, relative humidity, time-series study

## Abstract

**Background:**

Fungal otitis externa is generally regarded as more common in warm and humid environments, yet long-term daily time-series evidence on short-term associations with meteorological conditions remains limited.

**Objective:**

To examine short-term associations between daily meteorological factors and outpatient visits for clinically diagnosed fungal otitis externa, with particular attention to non-linear and lagged patterns.

**Methods:**

This single-center retrospective time-series study used outpatient registry data from Wuxi Huishan District People's Hospital in eastern China, covering January 1, 2014, to December 31, 2025. Daily outpatient visits were linked to meteorological data obtained from Open-Meteo. Candidate meteorological variables were screened using single-exposure generalized additive models, and the main associations were evaluated using distributed lag non-linear models within a quasi-Poisson framework, with adjustment for long-term temporal trend and weekday effects. Cumulative relative risks (RRs) and 95% confidence intervals (CIs) were estimated using the median exposure level as the reference. Collinearity among exposures was assessed using Spearman correlation.

**Results:**

A total of 6,260 outpatient visits were identified over 4,383 observation days, with a mean daily count of 1.43 visits. Visits were concentrated from July to October. In the main models, relative humidity and precipitation showed the most consistent cumulative associations with outpatient visits. Compared with the median level, the cumulative RR for relative humidity was 1.236 (95% CI, 1.052–1.452) at the 90th percentile and 1.253 (95% CI, 1.038–1.514) at the 95th percentile. The corresponding cumulative RRs for precipitation were 1.087 (95% CI, 1.014–1.166) and 1.142 (95% CI, 1.022–1.276). These associations were concentrated mainly on lag days 2–5. Exploratory analyses suggested that the precipitation association may partly reflect an overlapping moisture-related environmental signal.

**Conclusions:**

Higher relative humidity and precipitation were associated with increased outpatient visits for clinically diagnosed fungal otitis externa. Moisture-related meteorological conditions may help characterize short-term fluctuations in outpatient burden.

## 1 Introduction

Fungal otitis externa is a fungal infection of the external auditory canal and is a common condition in routine otolaryngology practice (1–4). Patients typically present with pruritus, otalgia, aural fullness, hearing impairment or discomfort, otorrhea, or a sensation of a blocked ear (1–9). In some patients, the disease may follow a prolonged or recurrent course, often requiring repeated treatment and follow-up (2–4). Fungal otitis externa is generally considered more common in warm and humid environments, particularly when local or behavioral factors increase moisture retention or disrupt the epithelial barrier of the external auditory canal, such as swimming or other water exposure, excessive ear manipulation or instrumentation, topical antibiotic or steroid use, and altered cerumen conditions (1–3, 5–7, 10).

The underlying biology of fungal otitis externa also supports a role for moisture-related environmental exposure (1, 2, 5). Fungal growth in the external auditory canal may be facilitated by warm and moist conditions, loss or alteration of protective cerumen, local changes in canal pH and flora, and injury to the epithelial lining (1, 2, 5). Elevated humidity and water exposure may therefore create conditions favorable to fungal colonization, persistence, and symptom progression (1–3, 5–7, 10). Several clinical studies from China have described the manifestations and management of fungal otitis externa in hot, humid climates, particularly in southern regions (6, 9). From this perspective, meteorological indicators of environmental moisture, particularly relative humidity and precipitation, may be relevant to fluctuations in the outpatient burden of this condition.

Nevertheless, quantitative evidence linking daily meteorological exposure to short-term outpatient visits for fungal otitis externa remains limited. Most published studies on fungal otitis externa have focused on clinical manifestations, causative fungi, predisposing factors, diagnostic approaches, or treatment outcomes rather than on short-term variation in healthcare use (1–10). By comparison, time-series analyses have been used to investigate several related otolaryngologic conditions (11–13). Together with broader evidence that climate and weather variability may influence fungal disease ecology and exposure risk, these findings support the need to investigate weather-sensitive patterns in fungal otitis externa (14–16).

This gap is particularly relevant in eastern China. Wuxi experiences warm, rainy summers and relatively high ambient humidity, all of which may favor fungal growth in the external auditory canal and influence healthcare-seeking patterns for ear symptoms. However, long-term daily outpatient data from humid subtropical settings remain limited, and few studies have systematically evaluated both immediate and lagged meteorological effects on clinically diagnosed fungal otitis externa.

Therefore, we conducted a 12-year time-series study using outpatient registry data from a tertiary hospital in eastern China to investigate the short-term associations between daily meteorological factors and outpatient visits for clinically diagnosed fungal otitis externa, with particular attention to non-linear and lagged effects.

## 2 Materials and methods

### 2.1 Study design and setting

This single-center retrospective time-series study was conducted at Wuxi Huishan District People's Hospital, a tertiary hospital in eastern China. The study covered the period from January 1, 2014, to December 31, 2025, and aimed to evaluate the short-term associations between daily meteorological factors and outpatient visits for clinically diagnosed fungal otitis externa.

A time-series design was considered appropriate because both exposure and outcome data were available on a daily scale, allowing short-term temporal associations to be examined, including possible non-linear and lagged effects of meteorological conditions on outpatient burden (17–21). The study was reported in accordance with the Strengthening the Reporting of Observational Studies in Epidemiology (STROBE) statement (22).

### 2.2 Data sources and case identification

Outpatient visit data were retrieved from the hospital's standard outpatient registry system for the period from January 1, 2014, to December 31, 2025. Cases were identified based on routine outpatient diagnostic labels recorded by treating otolaryngologists. Eligible diagnostic labels included otomycosis, fungal otitis externa, Aspergillus-related otitis externa, and other fungal infections of the external auditory canal recorded in the registry. These labels correspond most closely to the International Classification of Diseases, Tenth Revision (ICD-10) category “otitis externa in mycoses.” Because case ascertainment was based on recorded clinical diagnoses rather than on a single standardized code-based algorithm, the study outcome was defined as clinically diagnosed fungal otitis externa rather than as a diagnosis based solely on coding rules. Within the ICD-10 framework, these conditions are most closely represented by H62.2^*^, with related index references including aspergillosis (B44.8†), candidiasis (B37.2†), and unspecified superficial mycosis (B36.9†).

As this registry-based retrospective study relied on routine clinical data, systematic mycological confirmation, including microscopy, fungal culture, and molecular testing, was unavailable. Accordingly, the outcome was defined as clinically diagnosed fungal otitis externa rather than laboratory-confirmed fungal otitis externa (8, 9).

The unit of analysis was the daily number of outpatient visits rather than the number of unique patients. All eligible visits recorded on the same calendar day were counted toward the daily outpatient burden. A complete daily sequence was generated for the entire study period, and days with no recorded outpatient visit for clinically diagnosed fungal otitis externa were coded as zero.

Because stable patient-level identifiers were not retained in the anonymized analytic dataset, a sensitivity analysis restricted to first visits or unique patients could not be performed. The outcome should therefore be interpreted as the daily outpatient visit burden for clinically diagnosed fungal otitis externa, rather than the incidence of newly diagnosed cases. This visit-based endpoint is clinically meaningful because it reflects real-world healthcare utilization and outpatient workload. In this context, repeated visits and follow-up consultations may have contributed to short-term variation in daily counts and should be considered when interpreting the estimated associations.

### 2.3 Meteorological exposure assessment

Daily meteorological data were obtained from Open-Meteo for the study hospital location in Wuxi and preprocessed into a unified analytic dataset covering the same period as the outpatient series (23). The dataset included daily mean, maximum, and minimum temperature; relative humidity; dew point; atmospheric pressure; mean wind speed; maximum gust speed; precipitation; rainfall; sunshine duration; and derived indicators, including diurnal temperature range, 24-h pressure change, proportional humidity, absolute humidity, and wind chill.

The merged analytic dataset therefore consisted of one record per day from January 1, 2014, to December 31, 2025, with corresponding daily outpatient counts and meteorological exposures. Because these exposure estimates reflected ambient meteorological conditions at the hospital location rather than individual-level exposure, some degree of exposure misclassification was unavoidable and may have attenuated the estimated associations.

In the main analyses, particular emphasis was placed on metrics for temperature, humidity, and precipitation because these variables were biologically plausible and potentially relevant to fungal growth and moisture retention in the external auditory canal. Based on the screening analyses and clinical interpretability, mean temperature, relative humidity, absolute humidity, and precipitation were selected for distributed lag non-linear modeling.

### 2.4 Outcome definition and study variables

The primary outcome was the daily number of outpatient visits for clinically diagnosed fungal otitis externa. This measure was used to reflect the real-world outpatient burden of disease in routine clinical practice rather than the incidence of microbiologically confirmed fungal infection.

Time-related covariates were derived from calendar date and included year, month, season, weekday, and a continuous time index representing the ordered sequence of observation days. Seasons were categorized as spring (March-May), summer (June-August), autumn (September-November), and winter (December-February). Weekday was modeled as a categorical variable to account for short-term variation in healthcare-seeking behavior and outpatient service organization.

Candidate meteorological exposures considered during model development included mean, maximum, and minimum temperature; relative humidity; dew point; absolute humidity; precipitation; atmospheric pressure; wind speed; and sunshine duration. Before model construction, these variables were summarized descriptively and examined for collinearity using pairwise Spearman correlation analyses (Supplementary Table S2; Supplementary Figure S5). Because several temperature- and moisture-related variables were moderately to strongly correlated, the main analyses were interpreted as exposure-specific associations that may partly reflect overlapping environmental signals rather than mutually independent effects. Relative humidity and precipitation were therefore emphasized in the main results as prespecified moisture-related exposures, whereas absolute humidity and mean temperature were retained as comparative exposures within the same modeling framework. The rationale for this selection was based on biological plausibility, screening-stage signal patterns, clinical interpretability, and consistency across sensitivity analyses, as summarized in Supplementary Table S3.

### 2.5 Statistical analysis

Daily outpatient visits for clinically diagnosed fungal otitis externa were used as the study outcome. Descriptive analyses were first performed to characterize the temporal distribution of outpatient burden over the study period. Total visits, mean, median, and maximum daily counts, and the proportion of zero-visit days were calculated. Annual, monthly, seasonal, and weekday distributions were also examined. Meteorological variables were summarized using means, standard deviations, medians, interquartile ranges, and minimum and maximum values, as appropriate. Exploratory Spearman correlation analyses were conducted to assess crude associations between daily outpatient counts and candidate meteorological variables.

Because the outcome was a count variable and showed mild-to-moderate overdispersion, time-series regression models were used for the formal association analyses (19–21). As an initial screening step, single-exposure generalized additive models were fitted using a quasi-Poisson distribution with a log link (18, 21). Candidate exposures included mean temperature, relative humidity, absolute humidity, precipitation, atmospheric pressure, wind speed, and sunshine duration. Several lag structures were evaluated for each exposure, including same-day exposure (lag 0), single-day lagged exposures (lags 1, 2, and 3), the average of lags 0–1, and the average of lags 0–3. Long-term temporal trend and seasonality were controlled using a smooth function of the time index, while weekday effects were entered as categorical covariates (18–21). Relative risks (RRs) and 95% confidence intervals (CIs) were estimated for each meteorological variable per interquartile-range increase. To assess the stability of the screening results, the same single-exposure models were re-fitted using negative binomial generalized additive models as a sensitivity analysis (21).

The main analyses were then performed using distributed lag non-linear models (DLNMs) to evaluate potentially non-linear and delayed associations between meteorological exposures and outpatient visits (17, 20, 24). Based on the screening analyses and clinical interpretability, mean temperature, relative humidity, absolute humidity, and precipitation were entered into separate distributed lag non-linear models. In the primary specification, the maximum lag period was set at 7 days. For most exposures, the exposure-response dimension was modeled with a natural cubic spline with 4 degrees of freedom, whereas the lag-response dimension was modeled with a natural cubic spline with 3 degrees of freedom (17, 20, 24). Because precipitation displayed a highly skewed distribution with a large proportion of zero values, a linear function was specified for its exposure-response dimension, whereas the lag dimension continued to be modeled with a natural spline (17, 24). All distributed lag non-linear models were fitted within a quasi-Poisson regression framework, with adjustment for long-term temporal trend and seasonality using natural cubic splines of time with 7 degrees of freedom per year, together with weekday effects (17–21, 24).

For the distributed lag non-linear analyses, the median exposure level (P50) was used as the reference value. Cumulative relative risks and 95% confidence intervals were estimated for selected exposure percentiles, particularly P90 versus P50 and P95 versus P50, which were treated as the principal contrasts of interest. Overall cumulative exposure-response curves and lag-response curves were generated to illustrate the shape and timing of the associations (17, 20, 24).

Sensitivity analyses were performed by varying key model parameters. Specifically, the degrees of freedom used to control for long-term temporal trend ranged from 6 to 8 per year, and the maximum lag period ranged from 5 to 10 days. For each model specification, cumulative relative risks for P90 versus P50 and P95 versus P50 were re-estimated. Directional consistency across these alternative specifications was used to assess the robustness of the observed associations (17, 20, 24).

Supplementary analyses were conducted to assess model adequacy, characterize lag-specific patterns, and examine whether moisture-related exposures reflected overlapping environmental signals. Model adequacy was evaluated using the proportion of zero-visit days, the variance-to-mean ratio, Pearson dispersion, residual autocorrelation function and partial autocorrelation function plots, and Ljung–Box tests for the main quasi-Poisson models (Supplementary Tables S4, S5; Supplementary Figure S6). To support interpretation of the short-lag pattern, lag-specific estimates for relative humidity and precipitation were summarized across lag days 0–7, with attention paid to the concentration of positive estimates across lag days 2–5 (Supplementary Table S6; Supplementary Figures S1, S2). Because relative humidity and precipitation may capture partially shared moisture-related environmental conditions, an exploratory lag-block sensitivity analysis was conducted using prespecified lag windows. In this analysis, lag days 2–5 were treated as the target window because the main DLNM results indicated that the positive associations were concentrated mainly during this period (Supplementary Table S7; Supplementary Figures S7, S8).

All statistical analyses were performed in R. Generalized additive models were fitted using the mgcv package, and distributed lag non-linear models were fitted using the dlnm package (17, 18). All tests were two-sided, and *P* < 0.05 was considered statistically significant.

### 2.6 Ethics statement

All data were anonymized before analysis and contained no personally identifiable information. The study was approved by the Ethics Committee of Wuxi Huishan District People's Hospital (Approval number: HYLL-Y-2025001) and was conducted in accordance with the Declaration of Helsinki and relevant ethical standards (25). Given the retrospective design and the use of de-identified outpatient registry data, the Ethics Committee waived the requirement for informed consent (25–27).

## 3 Results

### 3.1 Descriptive characteristics of outpatient visits

From January 1, 2014, to December 31, 2025, a total of 6,260 outpatient visits for clinically diagnosed fungal otitis externa were documented over 4,383 observation days. The mean daily visit count was 1.43, with a median of 1 and a maximum of 10 per day. Days without any recorded visits accounted for 31.21% of the study period, indicating that zero counts were common in the daily series, although the data remained suitable for time-series modeling (Table 1). The daily count series included 1,368 days with no recorded visits and showed mild-to-moderate overdispersion, as reflected by a variance-to-mean ratio of 1.50. These distributional features supported the use of count regression models allowing for extra-Poisson variability (Supplementary Table S4).

**Table 1:** Descriptive characteristics of daily outpatient visits for clinically diagnosed fungal otitis externa from 2014 to 2025.

Metric	Value
Study period	2014–01–01 to 2025–12–31
Observation days	4,383
Total outpatient visits	6,260
Daily visits, mean ± SD	1.43 ± 1.46
Daily visits, median (range)	1 (0–10)
Zero–visit days, n (%)	1,368 (31.21)
Variance–to–mean ratio	1.50

SD, standard deviation.

The variance was omitted from the main table to improve compactness; the variance–to–mean ratio was retained to summarize mild–to–moderate overdispersion in the daily count series.

Data are presented as counts, means, medians, or summary statistics, as appropriate.

### 3.2 Temporal distribution of outpatient burden

**Figure 1:**
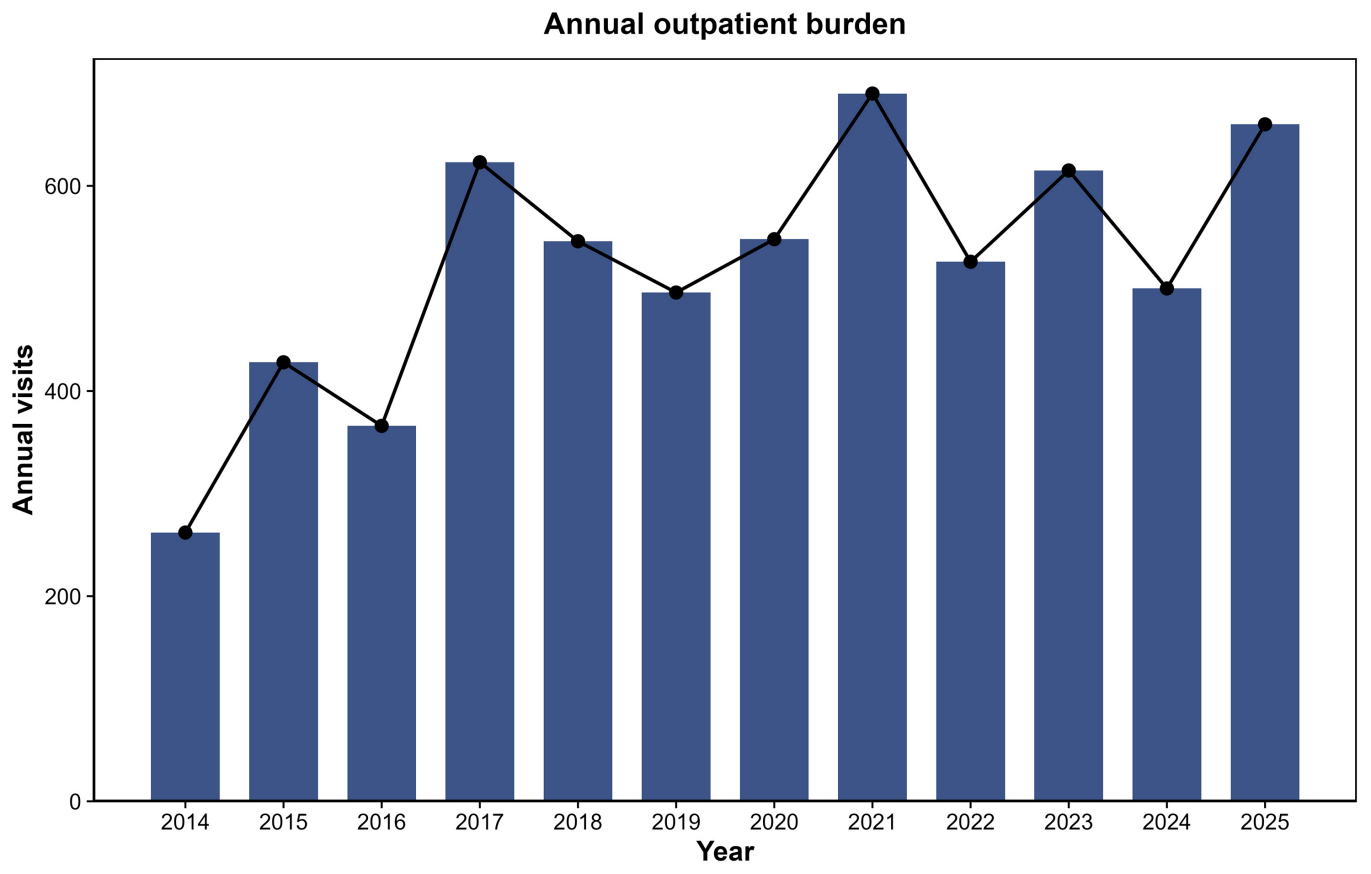
Annual outpatient burden of clinically diagnosed fungal otitis externa from 2014 to 2025. The annual number of outpatient visits for clinically diagnosed fungal otitis externa is shown for each calendar year. The bars represent yearly totals, and the superimposed line indicates the overall temporal trend across the study period.

**Figure 2:**
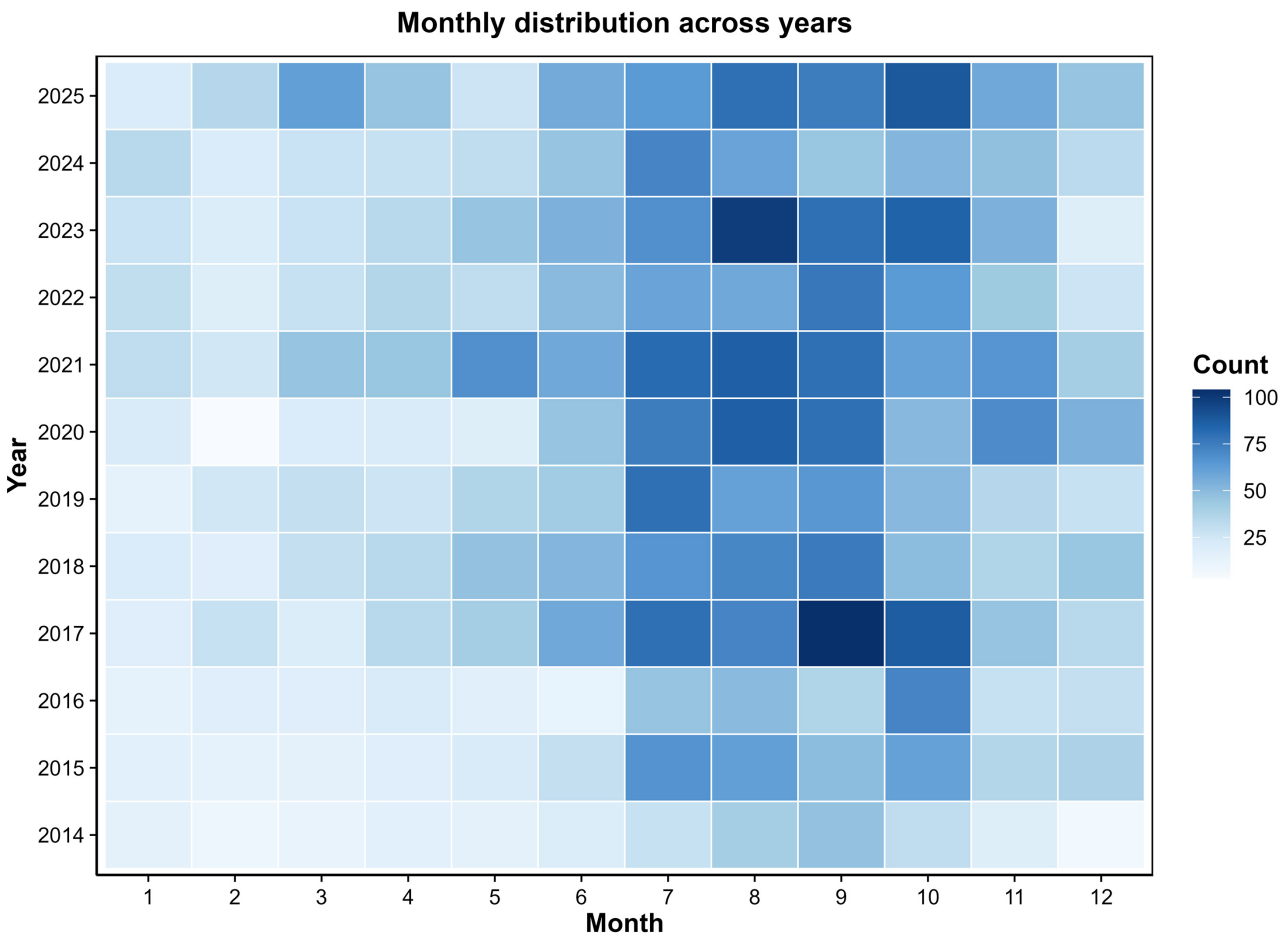
Monthly distribution of outpatient visits for clinically diagnosed fungal otitis externa from 2014 to 2025. Heatmap of monthly outpatient visits for clinically diagnosed fungal otitis externa from 2014 to 2025. Darker shading indicates a higher number of visits. Visits are more frequent during the warmer, more humid months, consistent with the seasonal pattern observed in this study.

The annual number of outpatient visits increased overall during the study period, though the pattern was characterized by year-to-year fluctuations rather than a steady, monotonic rise. Several of the higher-burden years occurred toward the end of the series (Figure 1). At the monthly level, visits were concentrated in the warmer and more humid months, with the highest mean daily counts occurring from July to October. September showed the greatest mean daily burden (2.26 visits/day), followed by August (2.22 visits/day), July (2.12 visits/day), and October (2.03 visits/day). A similar pattern was evident by season. Mean daily visit counts were 1.94 in summer and 1.93 in autumn, compared with 1.00 in spring and 0.84 in winter, indicating a clear concentration of outpatient burden during the warm seasons (Figure 2).

A modest weekday pattern was also present. The highest average daily counts were observed on Monday (1.66 visits/day) and Sunday (1.61 visits/day).

### 3.3 Single-exposure screening analysis

Exploratory correlation analysis indicated that the strongest positive crude correlations with daily outpatient visits were observed for temperature- and moisture-related variables, including minimum temperature, mean temperature, absolute humidity, and dew point. In contrast, atmospheric pressure was negatively correlated with visit counts. Because these exploratory analyses did not account for long-term trend, seasonality, or weekday effects, formal time-series models were subsequently fitted.

In the single-exposure generalized additive models, the largest effect estimates were observed for mean temperature at lag 0, absolute humidity at lag 3, and relative humidity at lag 3. The direction of association was generally consistent across the quasi-Poisson and negative binomial specifications, supporting the inclusion of these variables in the subsequent distributed lag non-linear analyses.

### 3.4 Main DLNM results

**Figure 3:**
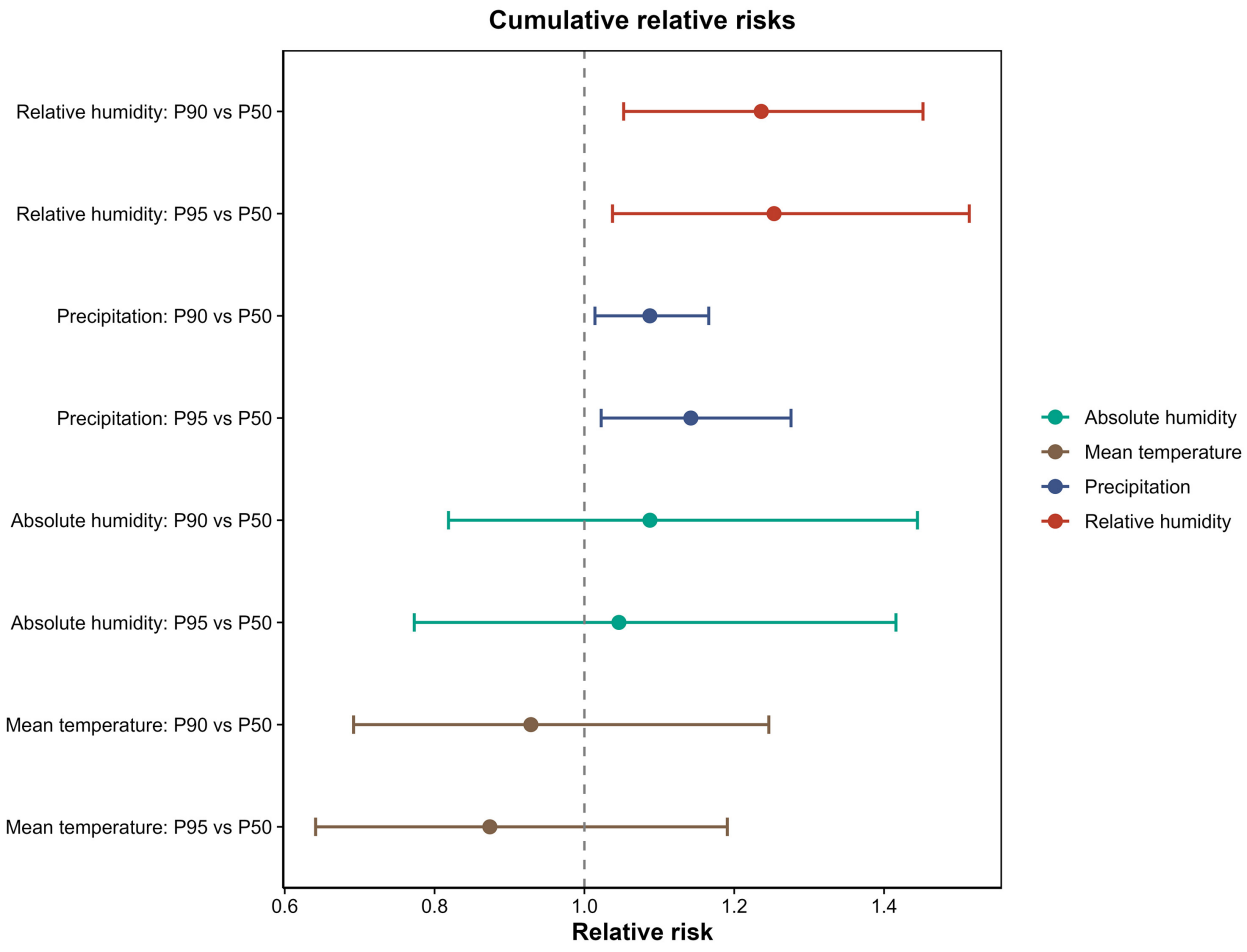
Cumulative relative risks under the main distributed lag non-linear model specification. Forest plot of cumulative relative risks for selected meteorological exposures under the main distributed lag non-linear model specification, using 7 degrees of freedom per year for long-term trend control and a maximum lag of 7 days. Effect estimates are presented for the P90 versus P50 and P95 versus P50 contrasts. Error bars indicate 95% confidence intervals. The horizontal dashed line indicates RR = 1.0.

**Figure 4:**
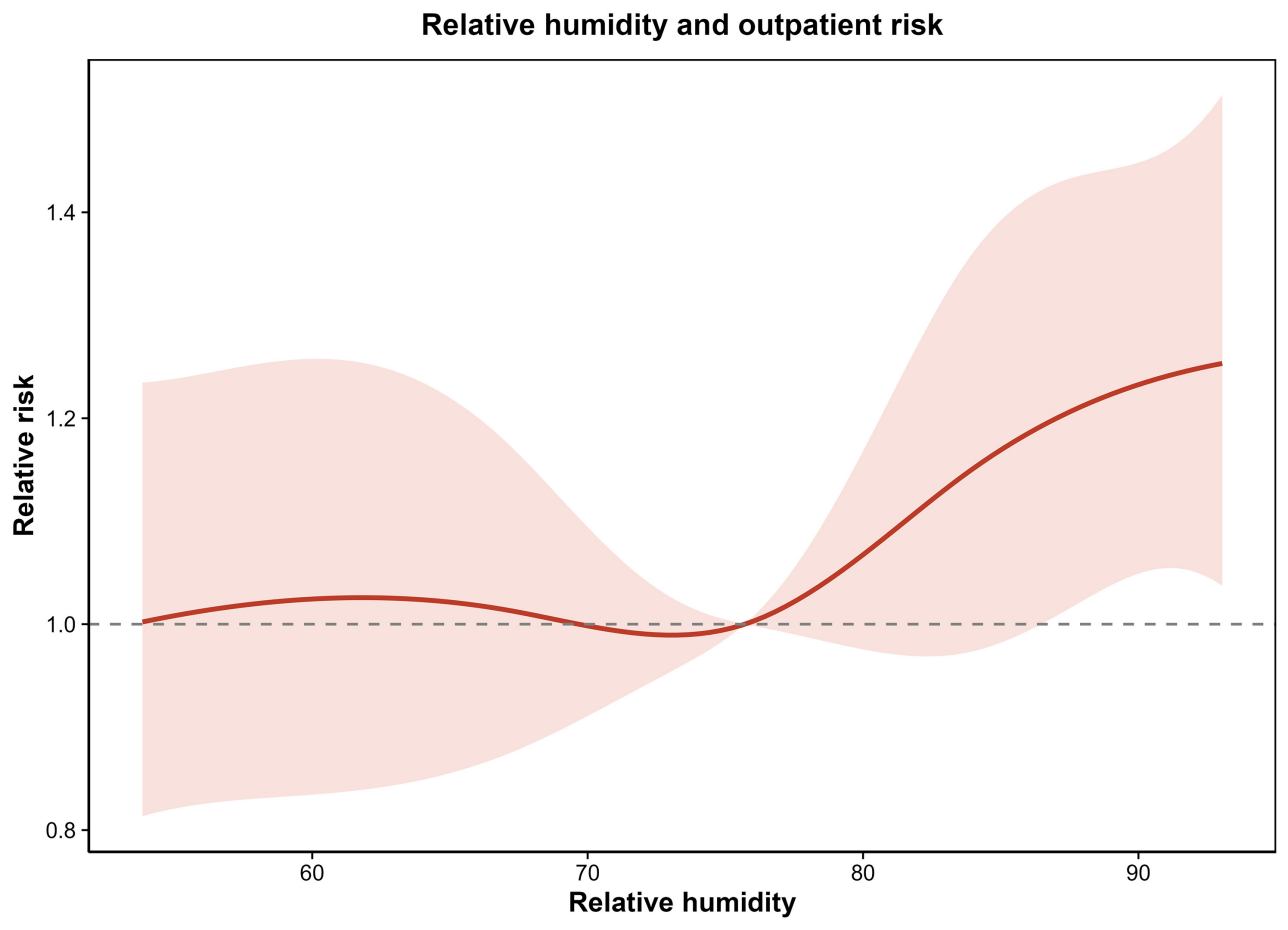
Relative humidity and outpatient visit risk. Overall cumulative exposure-response curve for relative humidity derived from the distributed lag non-linear model. The solid line represents the estimated cumulative relative risk of outpatient visits for clinically diagnosed fungal otitis externa across the observed range of relative humidity, using the median level as the reference. The shaded area indicates the 95% confidence interval. The horizontal dashed line indicates RR = 1.0.

**Figure 5:**
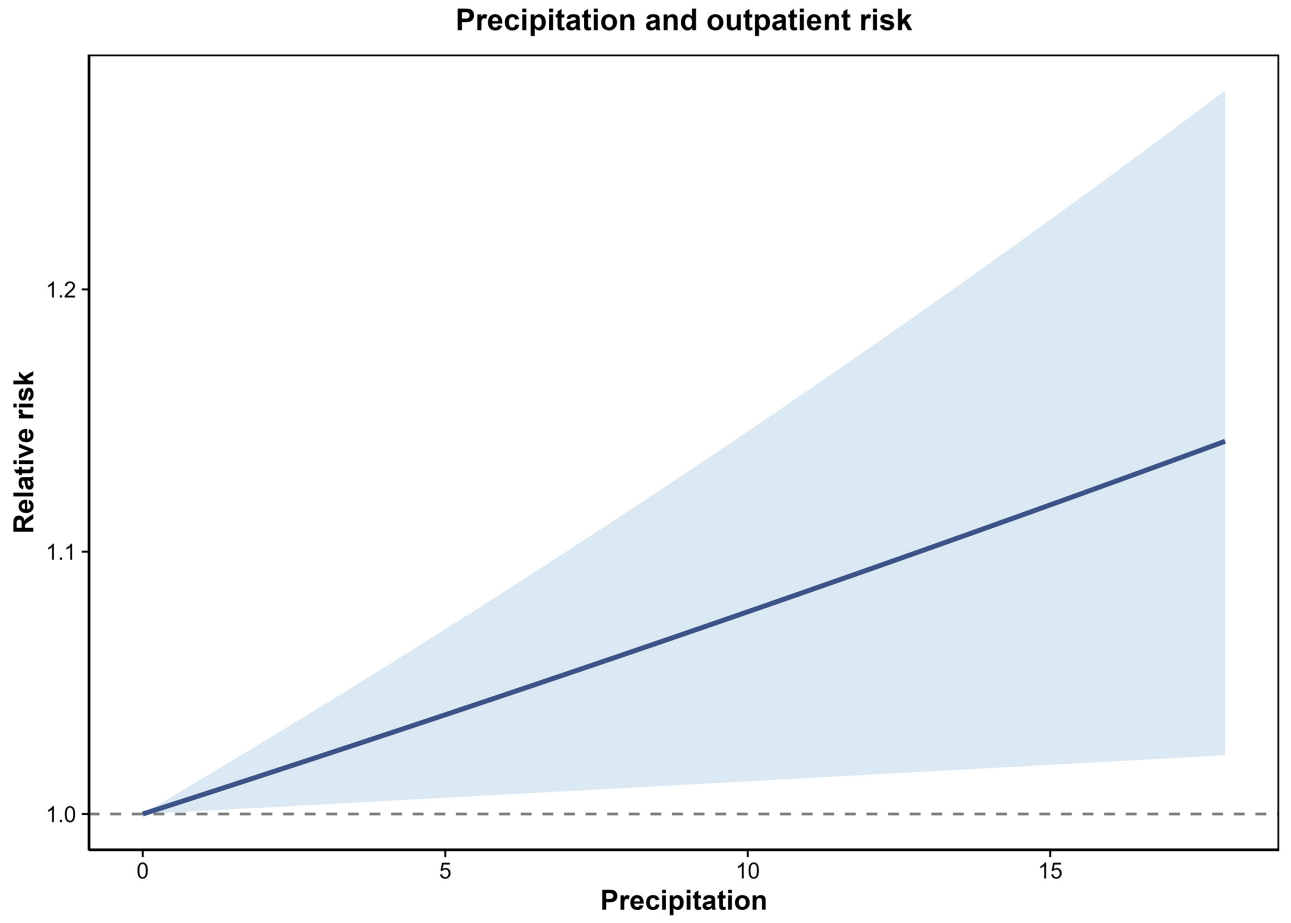
Precipitation and outpatient visit risk. Overall cumulative exposure-response curve for daily precipitation derived from the distributed lag non-linear model. The solid line represents the estimated cumulative relative risk of outpatient visits for clinically diagnosed fungal otitis externa across the observed precipitation range, with the median level as the reference. The shaded area indicates the 95% confidence interval. The horizontal dashed line indicates RR = 1.0.

Under the main model specification, which incorporated 7 degrees of freedom per year for long-term trend control and a maximum lag of 7 days, relative humidity and precipitation showed positive cumulative associations with outpatient visits for clinically diagnosed fungal otitis externa (Table 2, Figures 3–5).

**Table 2:** Main distributed lag non–linear model results for cumulative associations of meteorological exposures with outpatient visits for clinically diagnosed fungal otitis externa.

Variable	P50	P90	Cumulative RR (95% CI), P90 vs P50	P95	Cumulative RR (95% CI), P95 vs P50
Absolute humidity	11.43	23.21	1.087 (0.819–1.445)	24.20	1.046 (0.773–1.416)
Precipitation	0.00	11.28	1.087 (1.014–1.166)	17.89	1.142 (1.022–1.276)
Relative humidity	75.71	90.42	1.236 (1.052–1.452)	93.04	1.253 (1.038–1.514)
Mean temperature	18.05	29.13	0.929 (0.692–1.246)	30.86	0.874 (0.641–1.191)

Cumulative relative risks are presented for two prespecified contrasts, P90 versus P50 and P95 versus P50, for each exposure. P50 represents the median exposure level and was used as the reference. RR, relative risk; CI, confidence interval. Estimates were derived from the main distributed lag non–linear model with 7 degrees of freedom per year for long-term trend control and a maximum lag of 7 days.

For relative humidity, higher exposure levels were associated with an increased cumulative risk of outpatient visits. Relative to the median level (P50), the cumulative RR was 1.236 (95% CI, 1.052–1.452) at the 90th percentile and 1.253 (95% CI, 1.038–1.514) at the 95th percentile. The exposure-response curve indicated an upward gradient at higher humidity levels, while the lag-response curve suggested that the excess risk was concentrated primarily on lag days 2–5, with the greatest contribution around lag days 3–4.

Precipitation was also positively associated with cumulative risk. Compared with the median level, the cumulative RR was 1.087 (95% CI, 1.014–1.166) at the 90th percentile and 1.142 (95% CI, 1.022–1.276) at the 95th percentile. The corresponding lag-response pattern suggested that the excess risk was distributed mainly across lag days 2–5 rather than being limited to the same day.

Supplementary lag-specific analyses helped delineate the temporal pattern of the associations observed for the two primary moisture-related exposures. For relative humidity, the estimated relative risks were mainly above 1.0 across lag days 2–5, with the highest estimates observed around lag days 3–4. Precipitation showed a broadly similar short-lag pattern, although the lag-specific estimates were smaller in magnitude and should be interpreted with caution. Overall, these results were consistent with the delayed pattern observed in the cumulative distributed lag non-linear models (Supplementary Table S6; Supplementary Figures S1, S2).

Absolute humidity showed a generally positive exposure-response pattern (Supplementary Figure S3), although the cumulative associations were weaker and less precisely estimated. For instance, relative to the median level, the cumulative RR at the 95th percentile was 1.046 (95% CI, 0.773–1.416), indicating a positive association but with limited statistical precision.

Mean temperature, in contrast, did not demonstrate a consistent positive cumulative association in the main distributed lag non-linear models. Relative to the median level, the cumulative RR was 0.929 (95% CI, 0.692–1.246) at the 90th percentile and 0.874 (95% CI, 0.641–1.191) at the 95th percentile. The cumulative exposure-response curve did not show a clear monotonic rise in risk at higher temperatures (Supplementary Figure S4), suggesting that mean temperature was not consistently associated with outpatient burden within the present modeling framework.

### 3.5 Sensitivity analyses

Sensitivity analyses were conducted by varying the degrees of freedom used for long-term trend control (6, 7, and 8 per year) and the maximum lag period (5, 7, and 10 days). The positive associations observed for relative humidity and precipitation remained highly consistent across model specifications. For both variables, all nine model combinations produced effect estimates greater than 1.0 for the high-versus-median contrast, supporting the stability of the main findings (Supplementary Table S1).

Absolute humidity also yielded effect estimates above 1.0 across all model settings; however, the magnitude of association was smaller, and the confidence intervals were generally wider, indicating lower statistical stability than that observed for relative humidity or precipitation. Mean temperature showed greater variation in both direction and magnitude across sensitivity models and therefore was not retained as a primary exposure in the final interpretation.

Taken together, these analyses indicate that humidity-related exposures, particularly relative humidity, together with precipitation, showed stable associations, whereas mean temperature did not show a consistent pattern.

### 3.6 Exploratory lag-block sensitivity analyses of overlapping moisture-related signals

In the exploratory lag-block sensitivity analyses focused on the prespecified lag-day 2–5 window, the estimates for relative humidity remained above 1.0 after additional adjustment for rainy-day burden within the same lag window, although attenuation was observed. For the relative humidity lag-day 2–5 block, the RR was 1.096 (95% CI, 1.051–1.143) for the P90 versus P50 contrast and 1.119 (95% CI, 1.063–1.178) for the P95 versus P50 contrast in the main block model. After additional adjustment for rainy-day burden, the corresponding estimates were 1.071 (95% CI, 1.014–1.131) and 1.088 (95% CI, 1.018–1.163), respectively.

For precipitation, attenuation was more pronounced after adding relative humidity to the model, within the same lag window. In the main precipitation block model, the RR was 1.051 (95% CI, 1.017–1.086) for the P90 versus P50 contrast and 1.087 (95% CI, 1.028–1.149) for the P95 versus P50 contrast. After adjustment for relative humidity, these estimates decreased to 1.004 (95% CI, 0.961–1.048) and 1.006 (95% CI, 0.936–1.082), respectively (Supplementary Table S7; Supplementary Figures S7, S8). These exploratory findings suggest that the precipitation association may partly reflect an overlapping moisture-related environmental signal, rather than a clearly separable independent exposure, in the present dataset.

## 4 Discussion

### 4.1 Principal findings

In this 12-year single-center time-series study of outpatient visits for clinically diagnosed fungal otitis externa, higher relative humidity showed the most stable positive association with short-term outpatient visit burden, whereas greater precipitation was also associated with higher outpatient visit counts in the main distributed lag non-linear models. Mean temperature did not show a stable cumulative association in the main models, although formal comparisons of relative importance across meteorological exposures were not performed. The exploratory lag-block sensitivity analyses suggested that these two moisture-related exposures should be interpreted as partly overlapping environmental signals rather than as separate independent effects. In these analyses, the short-lag association for relative humidity remained above 1.0 after additional adjustment for rainy-day burden, though attenuated. By comparison, the precipitation estimates were markedly attenuated after adjustment for relative humidity within the same lag window (Supplementary Table S7; Supplementary Figures S7, S8).

### 4.2 Interpretation of the humidity- and precipitation-related associations

One of the main observations of the present study is that relative humidity and precipitation showed positive associations in the main distributed lag non-linear models, whereas mean temperature did not show a stable cumulative association; however, no formal comparison of relative importance among meteorological exposures was performed. This finding is biologically and clinically plausible. Fungal otitis externa is unlikely to be driven solely by heat; instead, it appears to depend more closely on a persistently damp microenvironment within the external auditory canal, which may facilitate fungal colonization, persistence, and symptom progression. Relative humidity may therefore provide a closer approximation to the environmental conditions that favor moisture retention within the ear canal, whereas precipitation may reflect broader moisture-related conditions, including increases in ambient dampness, prolonged wetting of the skin and hair, and moisture-related behaviors such as delayed drying of the external ear (1, 2, 4–6, 14–16).

The lag pattern also warrants consideration. For both relative humidity and precipitation, the excess risk was concentrated several days after exposure rather than being confined to the same day. Such a delay is biologically and clinically reasonable. Moisture-related environmental changes may take time to contribute to local microbial proliferation, debris accumulation, worsening of pruritus or blockage, and, ultimately, presentation for outpatient care. This pattern suggests that sustained damp conditions, rather than a brief transient fluctuation, may be more relevant to short-term outpatient burden.

These findings call for a cautious interpretation of the precipitation results. Rather than being interpreted as a clearly separable independent exposure, precipitation may partly reflect a broader moisture-related environmental signal that overlaps with the signal captured by relative humidity. This interpretation is consistent with the correlation pattern among candidate meteorological exposures and with the exploratory lag-block sensitivity analyses, in which the precipitation estimates were markedly attenuated after adjustment for relative humidity within the same lag window (Supplementary Figure S5, Supplementary Table S7, Supplementary Figures S7 and S8).

Mean temperature showed an association in the initial screening models but did not remain a stable primary exposure in the main distributed lag non-linear framework. This should not be taken to mean that temperature has no role. A more cautious interpretation is that its association may have been attenuated after temporal adjustment, may be partly shared with other meteorological characteristics, or may be less proximate to the pathophysiologic processes underlying fungal otitis externa than humidity-related exposures (1, 2, 4–6, 14–16).

### 4.3 Comparison with previous studies

The present findings are broadly consistent with the long-standing clinical view that fungal otitis externa occurs more frequently in warm and humid environments (1–3, 5–7, 10). Earlier studies have focused mainly on predisposing factors such as swimming, ear manipulation, topical medication use, and other conditions that increase local moisture within the external auditory canal (1–3, 5–7, 10). Against that background, the current study adds a temporal dimension by indicating that, at the daily level, moisture-related meteorological conditions were associated with short-term variation in outpatient burden.

This interpretation is also compatible with the broader literature on weather-sensitive otolaryngologic diseases. Previous investigations have described associations between extreme weather events and emergency visits for acute otitis externa, as well as between meteorological variables and outpatient visits for chronic rhinosinusitis (11–13). Although those studies concerned different disease entities, they support the broader proposition that day-to-day weather conditions may influence healthcare utilization in otolaryngology. A similar perspective has emerged from recent work on climate-sensitive fungal diseases, in which environmental risk has been linked not only to ambient temperature but also to moisture-related conditions, precipitation patterns, and ecological change (14–16). Considered together, these findings support a more differentiated understanding of environmental risk in fungal otitis externa.

### 4.4 Clinical and public health implications

The present findings may have implications for both clinical practice and outpatient service planning. For otolaryngologists, sustained high humidity or increased rainfall may indicate periods when outpatient demand is more likely to increase. Under such circumstances, earlier patient education regarding ear drying, avoidance of unnecessary instrumentation of the external canal, and careful management after swimming, bathing, or prolonged moisture exposure may be justified. At the service level, the findings may also help anticipate short-term or seasonal increases in clinic burden, thereby informing staffing and scheduling during higher-risk periods. More broadly, moisture-related weather conditions may serve as a useful environmental signal for anticipating fluctuations in clinically diagnosed fungal otitis externa in routine outpatient practice.

### 4.5 Strengths and limitations

Several features of the study strengthen the interpretation of the findings. First, the 12-year continuous daily dataset reduced the influence of short-term anomalies and enabled more stable estimation of temporal patterns. Second, the daily time-series design enabled examination not only of whether meteorological factors were associated with outpatient burden, but also of when those associations emerged. Third, the use of distributed lag non-linear models allowed for the evaluation of non-linear and delayed associations within a single analytic framework, which is well suited to weather-related outpatient data (17, 20, 24). Fourth, using daily outpatient visits as the endpoint is directly interpretable in clinical practice because it reflects real-world healthcare utilization and outpatient workload, rather than incident disease alone. The moisture-related findings were further assessed through supplementary analyses, including alternative specifications for long-term temporal trend control and the maximum lag period, lag-specific summaries, and exploratory assessments of overlapping moisture-related environmental signals (Supplementary Table S1, Supplementary Tables S6, S7, Supplementary Figures S1, S2, S7, S8). Finally, the endpoint itself—clinically diagnosed fungal otitis externa in a tertiary otolaryngology setting—has direct clinical relevance and clear practical interpretability.

Several limitations should also be considered. The outcome was based on routine outpatient registry diagnoses rather than mycological confirmation; accordingly, the findings reflect the outpatient burden of clinically diagnosed disease in routine practice rather than the incidence of laboratory-confirmed fungal otitis externa. Some degree of diagnostic misclassification is therefore unavoidable, particularly because fungal and bacterial otitis externa may overlap clinically. Even so, this limitation does not materially diminish the study's practical value, since the research question concerned real-world outpatient burden rather than pathogen-specific microbiological epidemiology.

The single-center design may also limit generalizability to other climatic regions and healthcare settings. Moreover, the outcome was defined based on outpatient visits rather than unique incident cases, and the observed temporal distribution may therefore have been influenced by healthcare-seeking behavior. Because stable patient-level identifiers were not retained in the anonymized analytic dataset, repeated visits could not be reliably distinguished from first visits. The findings should therefore be interpreted as temporal patterns in outpatient visit burden rather than as estimates of incident cases at the individual-patient level. Repeated visits by the same patient, together with unmeasured time-varying factors such as service organization or holiday effects, may also have contributed to short-term fluctuation. Meteorological exposures were estimated from ambient conditions at the hospital location rather than from individual-level measurements. Therefore, some exposure misclassification was unavoidable. This limitation may have attenuated the estimated associations. Although adjustments were made for temporal trends and weekday effects, and multiple sensitivity analyses were performed, residual confounding cannot be completely excluded.

## 5 Conclusion

In this 12-year single-center time-series study, higher relative humidity was associated with increased outpatient visits for clinically diagnosed fungal otitis externa, whereas mean temperature did not show a stable cumulative association in the main distributed lag non-linear models. Precipitation also showed a positive association with outpatient visits in the main models, although supplementary analyses suggested that this association may partly reflect an overlapping moisture-related environmental signal rather than a clearly separable independent exposure. These findings should therefore be interpreted as showing that moisture-related meteorological conditions are associated with short-term fluctuations in outpatient visit burden, rather than as evidence of causal effects on disease occurrence. In this context, clinically diagnosed fungal otitis externa may be regarded as a moisture-sensitive outpatient condition in routine otolaryngology practice. These results may inform careful consideration of weather-informed prevention strategies, patient education, and outpatient service planning. Further multicenter prospective studies with standardized diagnostic criteria and mycological confirmation are warranted to validate these associations.

## Data Availability

The raw data supporting the conclusions of this article will be made available by the authors, without undue reservation.
