# Supplementary material for: Relative humidity, precipitation, and outpatient visits for clinically diagnosed fungal otitis externa: evidence from a single-center time-series study in Wuxi, eastern China
Source: Front Public Health. 2026 Jun 19;14:1859702. doi: 10.3389/fpubh.2026.1859702 (PMC13328466; doi:10.3389/fpubh.2026.1859702)
Supplement: Supplementary file 1 [file Data_Sheet_1.pdf]

Supplementary materials

Relative Humidity, Precipitation, and Outpatient Visits for Clinically Diagnosed Fungal Otitis Externa: Evidence From a Single-Center Time-Series Study in Wuxi, Eastern China

Supplementary Figure S1. Lag-specific relative risks for relative humidity.

Lag-specific relative risks for the high-exposure contrast of relative humidity, comparing the 95th percentile with the median level (P95 versus P50). The solid line represents the estimated relative risk at each lag day, and the shaded area indicates the 95% confidence interval. The horizontal dashed line indicates a relative risk of 1.0. The positive association was concentrated mainly across lag days 2–5, with the highest estimates observed around lag days 3–4.

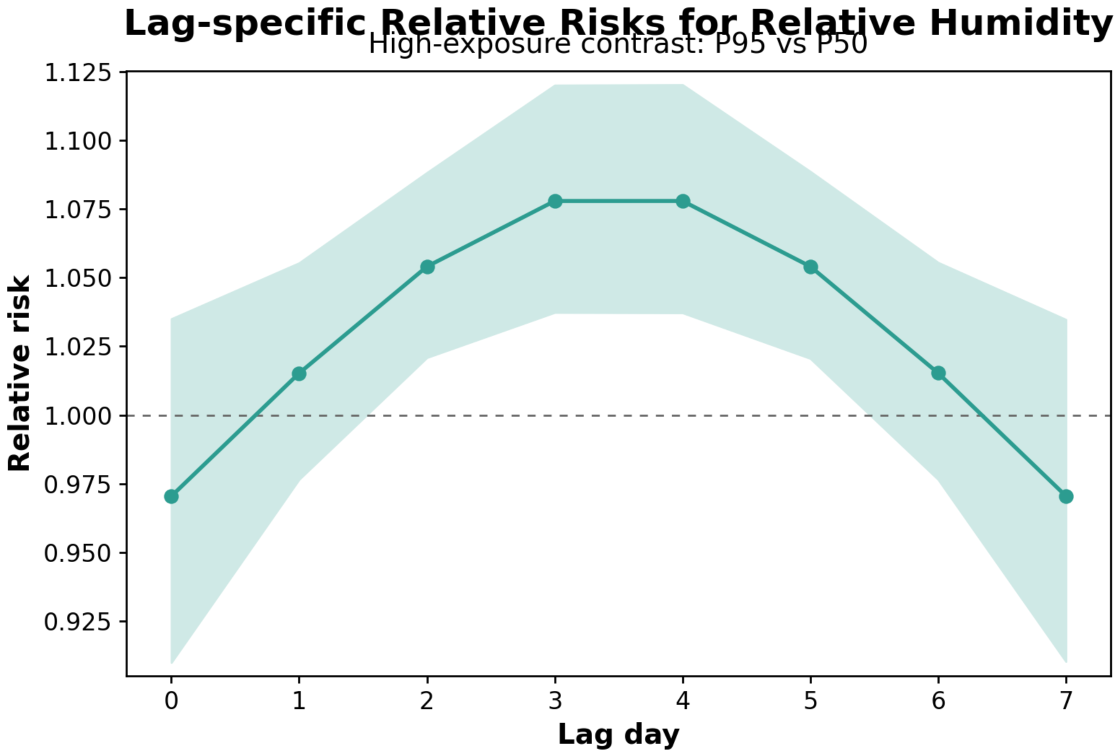

Supplementary Figure S2. Lag-specific relative risks for precipitation.

Lag-specific relative risks for high precipitation exposure, comparing the 95th percentile with the median level (P95 versus P50), across lag days 0–7. The solid line represents the estimated relative risk at each lag day, and the shaded area indicates the 95% confidence interval. The horizontal dashed line indicates a relative risk of 1.0. The lag-specific estimates were slightly elevated, particularly on lag days 2–5; however, they should be interpreted with caution given the relatively wide confidence intervals.

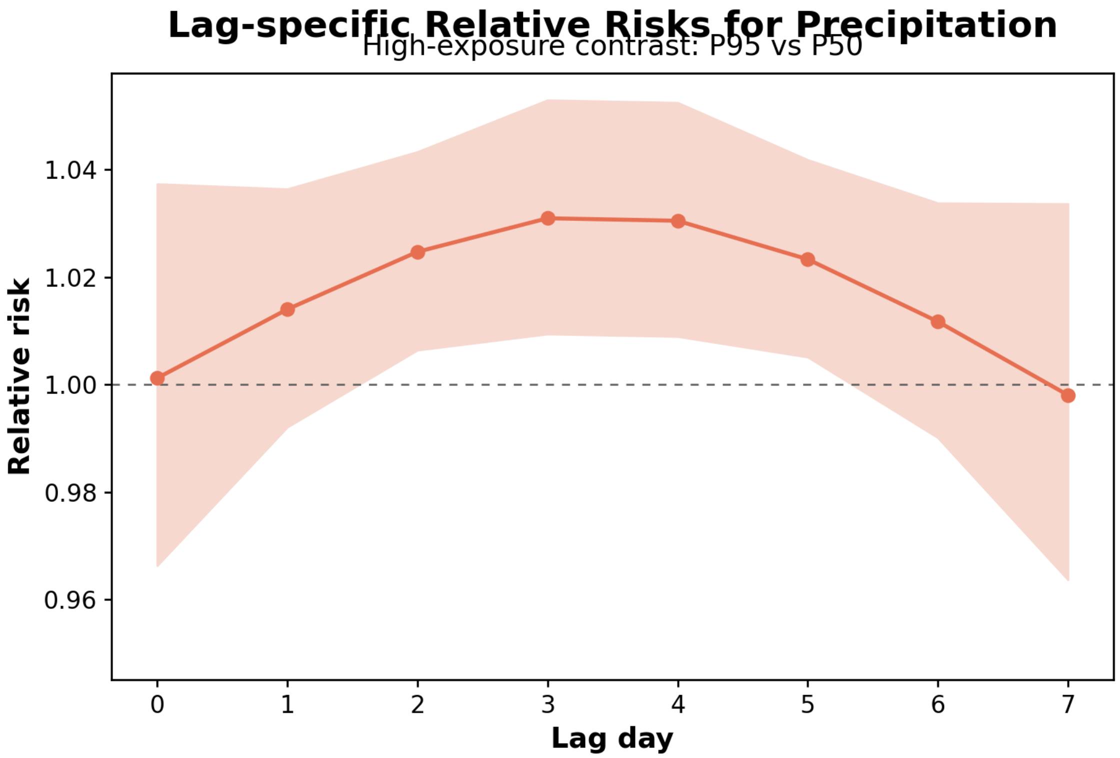

Supplementary Figure S3. Overall cumulative exposure-response curve for absolute humidity.

Overall cumulative exposure-response relationship between absolute humidity and outpatient visits for clinically diagnosed fungal otitis externa. The solid line represents the estimated cumulative relative risk, and the shaded area indicates the 95% confidence interval. The horizontal dashed line indicates a cumulative relative risk of 1.0. The curve suggests a generally positive pattern at higher absolute humidity levels, although the wide confidence intervals indicate limited statistical precision.

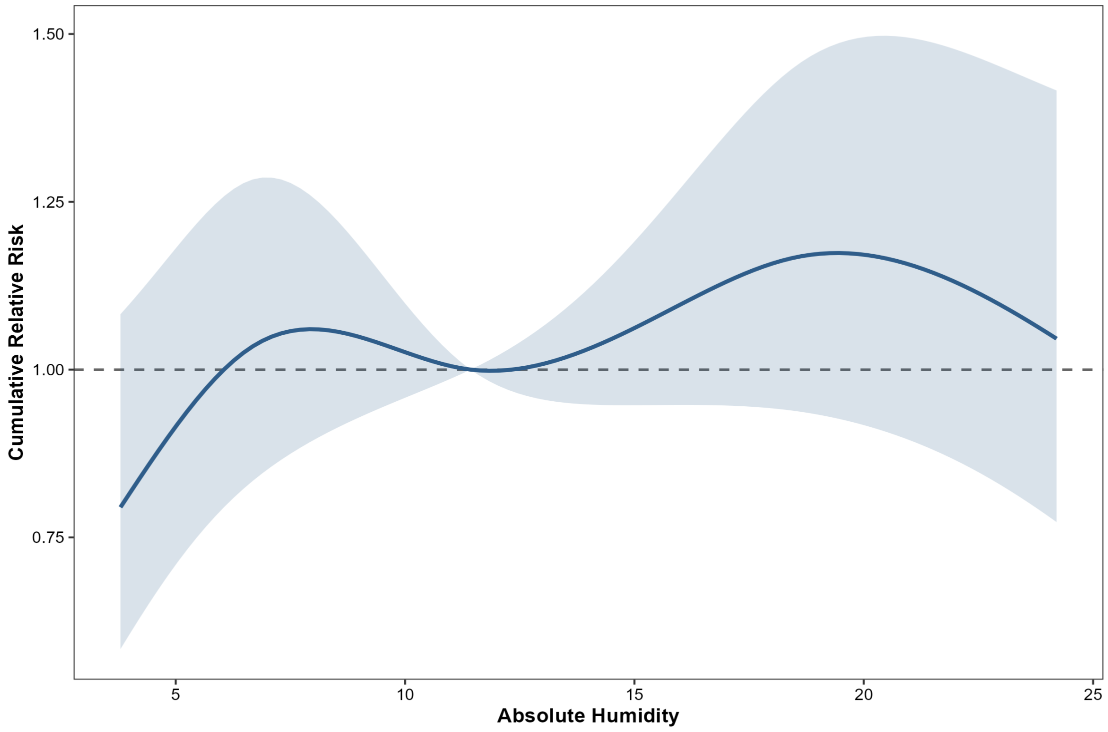

Supplementary Figure S4. Overall cumulative exposure-response curve for mean temperature.

Overall cumulative exposure-response relationship between mean temperature and outpatient visits for clinically diagnosed fungal otitis externa. The solid line represents the estimated cumulative relative risk, and the shaded area indicates the 95% confidence interval. The vertical dotted line indicates the centering value, corresponding to the median exposure level (P50), and the horizontal dashed line indicates a cumulative relative risk of 1.0. No clear monotonic increase in cumulative risk was observed at higher mean temperatures.

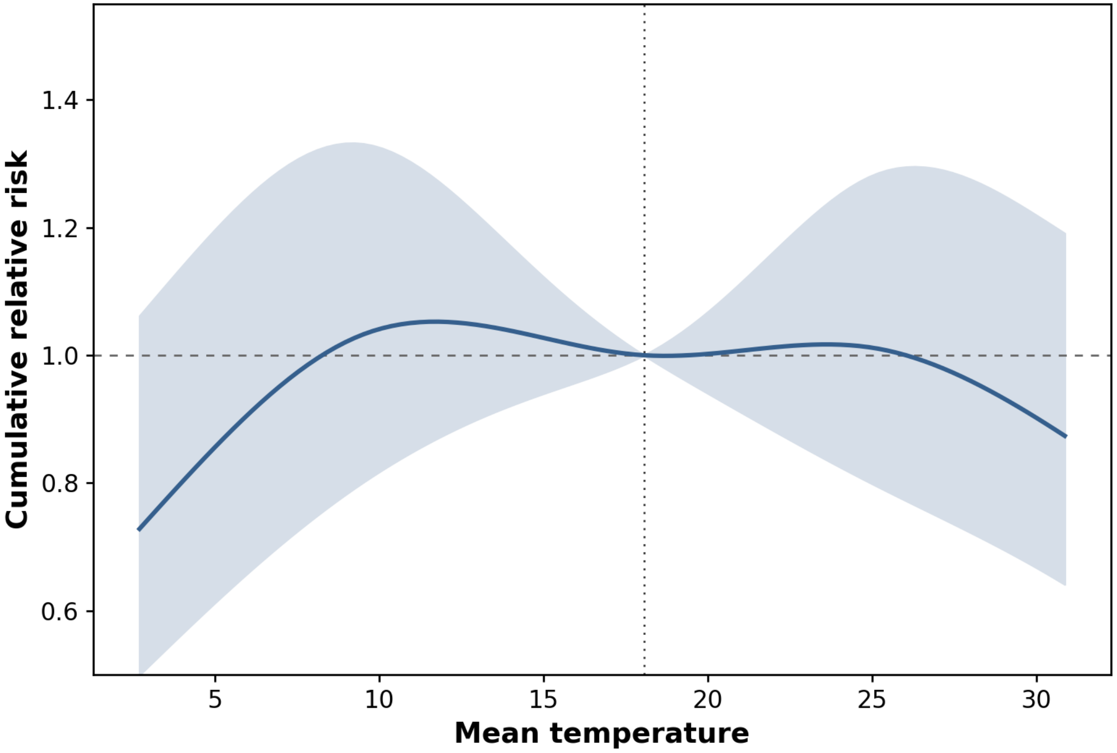

Supplementary Figure S5. Spearman correlation heatmap among candidate meteorological exposures.

Pairwise Spearman correlation coefficients among candidate meteorological variables are shown to assess collinearity before model construction. Red indicates a positive correlation, blue indicates a negative correlation, and darker colors indicate a stronger correlation. The heatmap shows strong correlations among temperature-related variables, dew point, and absolute humidity, whereas relative humidity and precipitation represented partly overlapping but not identical moisture-related exposure patterns.

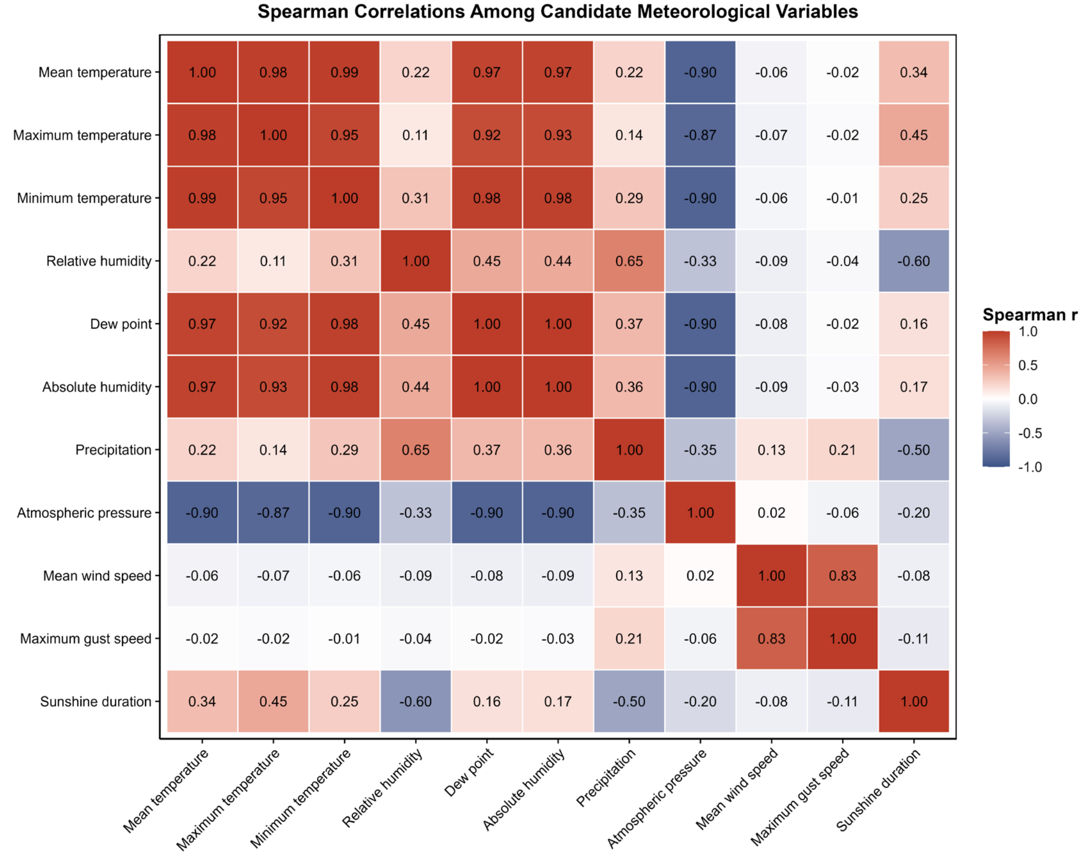

Supplementary Figure S6. Residual autocorrelation diagnostics for the main quasi-Poisson distributed lag non-linear models.

Pearson residual autocorrelation function (ACF) and partial autocorrelation function (PACF) plots are shown for the main quasi-Poisson distributed lag non-linear models of mean temperature, relative humidity, absolute humidity, and precipitation. The dashed blue lines indicate the approximate 95% reference bounds for residual autocorrelation. Although several isolated partial autocorrelation spikes were observed, no strong or persistent residual autocorrelation pattern was evident, supporting the overall adequacy of the temporal adjustment in the main models.

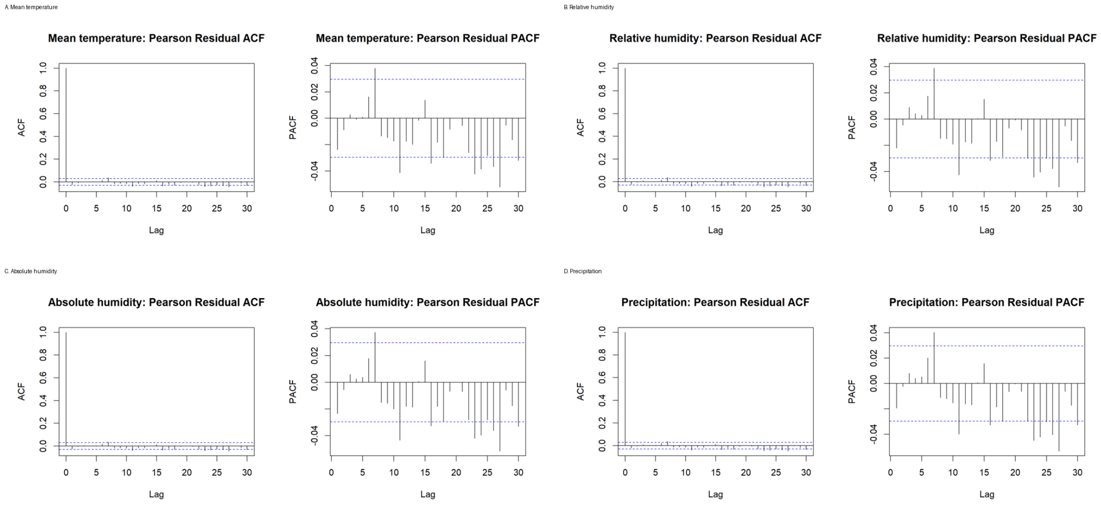

Supplementary Figure S7. Relative humidity lag days 2–5: main model versus rainy-day-adjusted model.

Point estimates and 95% confidence intervals are shown for the relative humidity block across lag days 2–5 in the exploratory shared-signal sensitivity analysis. The main model was compared with a model additionally adjusted for rainy-day burden within the same lag window. For both the P90 versus P50 and P95 versus P50 contrasts, the point estimates remained above 1.0 after adjustment, though attenuated. The vertical dashed line indicates a relative risk of 1.0.

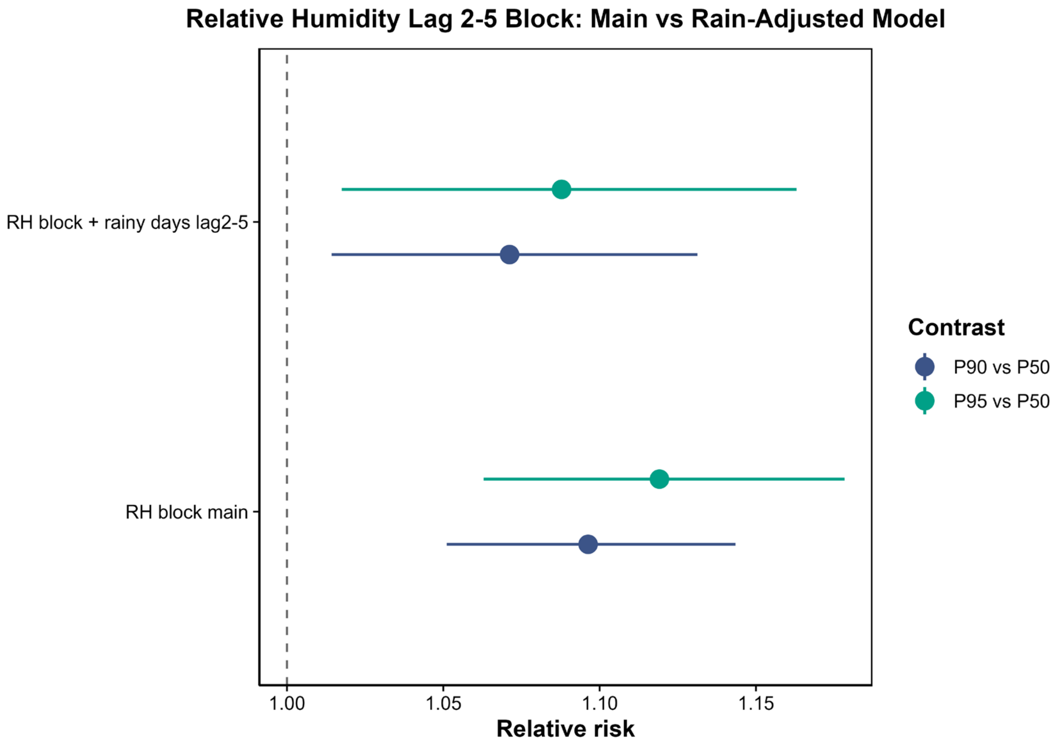

Supplementary Figure S8. Precipitation lag days 2–5: main model versus relative humidity-adjusted model.

Point estimates and 95% confidence intervals are shown for the lag days 2–5 precipitation block in the exploratory shared-signal sensitivity analysis. The main model was compared with a model additionally adjusted for relative humidity within the same lag window. After adjustment for relative humidity, the precipitation estimates were markedly attenuated and moved closer to the null, suggesting that the precipitation association may partly reflect overlapping moisture-related environmental conditions. The vertical dashed line indicates a relative risk of 1.0.

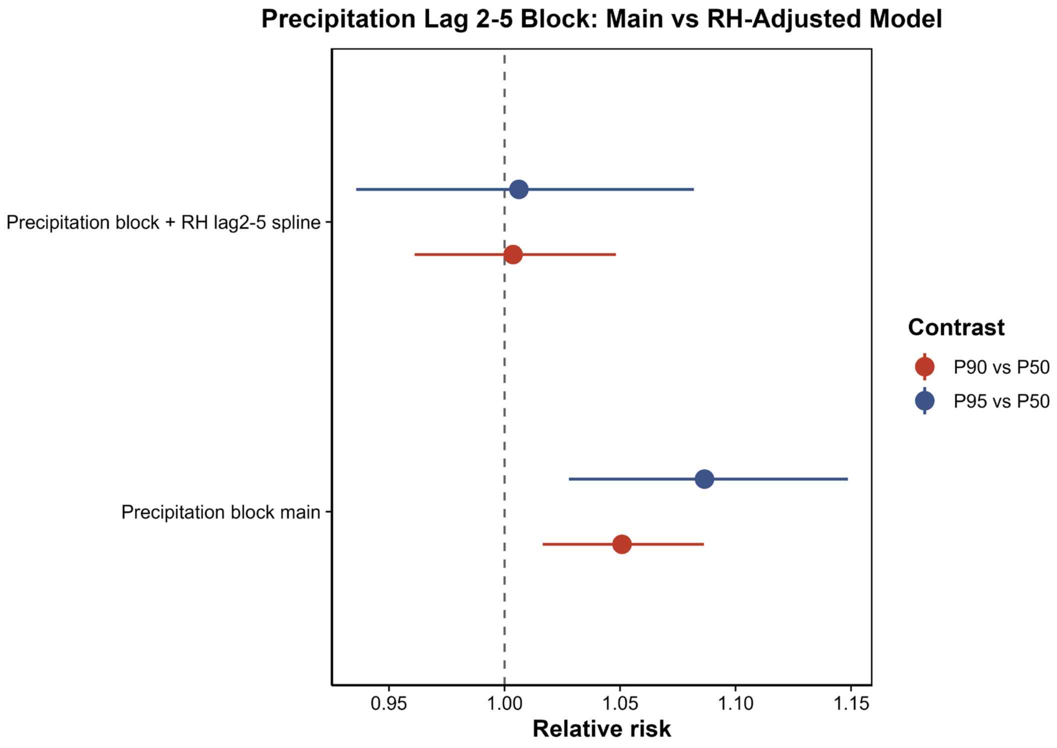

2. Supplementary Tables

Supplementary Table S1. Sensitivity analyses of cumulative associations of meteorological exposures with outpatient visits for clinically diagnosed fungal otitis externa under alternative temporal trend and lag specifications

Exposure	Contrast	Trend df/year	Max lag	Cumulative RR (95% CI)
Mean temperature	P90 vs P50	6	5	0.996 (0.770–1.289)
Mean temperature	P95 vs P50	6	5	0.979 (0.745–1.287)
Mean temperature	P90 vs P50	6	7	0.934 (0.701–1.244)
Mean temperature	P95 vs P50	6	7	0.909 (0.670–1.233)
Mean temperature	P90 vs P50	6	10	0.797 (0.574–1.105)
Mean temperature	P95 vs P50	6	10	0.755 (0.532–1.071)
Mean temperature	P90 vs P50	7	5	0.987 (0.759–1.282)
Mean temperature	P95 vs P50	7	5	0.940 (0.713–1.240)
Mean temperature	P90 vs P50	7	7	0.929 (0.692–1.246)
Mean temperature	P95 vs P50	7	7	0.874 (0.641–1.191)
Mean temperature	P90 vs P50	7	10	0.820 (0.582–1.155)
Mean temperature	P95 vs P50	7	10	0.743 (0.519–1.063)
Mean temperature	P90 vs P50	8	5	1.068 (0.816–1.399)
Mean temperature	P95 vs P50	8	5	1.048 (0.784–1.400)
Mean temperature	P90 vs P50	8	7	1.010 (0.745–1.369)
Mean temperature	P95 vs P50	8	7	0.984 (0.710–1.363)
Mean temperature	P90 vs P50	8	10	0.891 (0.623–1.273)
Mean temperature	P95 vs P50	8	10	0.839 (0.573–1.229)
Absolute humidity	P90 vs P50	6	5	1.230 (0.961–1.573)

Exposure	Contrast	Trend df/year	Max lag	Cumulative RR (95% CI)
Absolute humidity	P95 vs P50	6	5	1.228 (0.950–1.586)
Absolute humidity	P90 vs P50	6	7	1.121 (0.852–1.475)
Absolute humidity	P95 vs P50	6	7	1.116 (0.839–1.484)
Absolute humidity	P90 vs P50	6	10	1.129 (0.824–1.547)
Absolute humidity	P95 vs P50	6	10	1.114 (0.803–1.544)
Absolute humidity	P90 vs P50	7	5	1.204 (0.935–1.551)
Absolute humidity	P95 vs P50	7	5	1.166 (0.890–1.527)
Absolute humidity	P90 vs P50	7	7	1.087 (0.819–1.445)
Absolute humidity	P95 vs P50	7	7	1.046 (0.773–1.416)
Absolute humidity	P90 vs P50	7	10	1.083 (0.780–1.503)
Absolute humidity	P95 vs P50	7	10	1.020 (0.720–1.445)
Absolute humidity	P90 vs P50	8	5	1.214 (0.932–1.580)
Absolute humidity	P95 vs P50	8	5	1.206 (0.912–1.595)
Absolute humidity	P90 vs P50	8	7	1.089 (0.808–1.469)
Absolute humidity	P95 vs P50	8	7	1.083 (0.790–1.485)
Absolute humidity	P90 vs P50	8	10	1.097 (0.773–1.558)
Absolute humidity	P95 vs P50	8	10	1.076 (0.744–1.555)
Relative humidity	P90 vs P50	6	5	1.162 (1.008–1.338)
Relative humidity	P95 vs P50	6	5	1.178 (1.000–1.387)
Relative humidity	P90 vs P50	6	7	1.161 (0.990–1.360)
Relative humidity	P95 vs P50	6	7	1.164 (0.965–1.404)
Relative humidity	P90 vs P50	6	10	1.248 (1.038–1.501)

Exposure	Contrast	Trend df/year	Max lag	Cumulative RR (95% CI)
Relative humidity	P95 vs P50	6	10	1.190 (0.951–1.490)
Relative humidity	P90 vs P50	7	5	1.227 (1.063–1.416)
Relative humidity	P95 vs P50	7	5	1.255 (1.065–1.479)
Relative humidity	P90 vs P50	7	7	1.236 (1.052–1.452)
Relative humidity	P95 vs P50	7	7	1.253 (1.038–1.514)
Relative humidity	P90 vs P50	7	10	1.350 (1.118–1.629)
Relative humidity	P95 vs P50	7	10	1.301 (1.037–1.631)
Relative humidity	P90 vs P50	8	5	1.169 (1.008–1.355)
Relative humidity	P95 vs P50	8	5	1.190 (1.004–1.411)
Relative humidity	P90 vs P50	8	7	1.162 (0.982–1.375)
Relative humidity	P95 vs P50	8	7	1.170 (0.960–1.426)
Relative humidity	P90 vs P50	8	10	1.258 (1.030–1.538)
Relative humidity	P95 vs P50	8	10	1.201 (0.943–1.529)
Precipitation	P90 vs P50	6	5	1.070 (1.007–1.136)
Precipitation	P95 vs P50	6	5	1.113 (1.011–1.224)
Precipitation	P90 vs P50	6	7	1.066 (0.993–1.144)
Precipitation	P95 vs P50	6	7	1.107 (0.989–1.238)
Precipitation	P90 vs P50	6	10	1.065 (0.978–1.159)
Precipitation	P95 vs P50	6	10	1.104 (0.965–1.264)
Precipitation	P90 vs P50	7	5	1.088 (1.025–1.155)
Precipitation	P95 vs P50	7	5	1.143 (1.040–1.256)
Precipitation	P90 vs P50	7	7	1.087 (1.014–1.166)

Exposure	Contrast	Trend df/year	Max lag	Cumulative RR (95% CI)
Precipitation	P95 vs P50	7	7	1.142 (1.022–1.276)
Precipitation	P90 vs P50	7	10	1.089 (1.001–1.184)
Precipitation	P95 vs P50	7	10	1.144 (1.002–1.307)
Precipitation	P90 vs P50	8	5	1.068 (1.003–1.136)
Precipitation	P95 vs P50	8	5	1.109 (1.005–1.224)
Precipitation	P90 vs P50	8	7	1.059 (0.984–1.140)
Precipitation	P95 vs P50	8	7	1.095 (0.975–1.232)
Precipitation	P90 vs P50	8	10	1.049 (0.958–1.148)
Precipitation	P95 vs P50	8	10	1.078 (0.934–1.245)

Note*:* Cumulative relative risks are presented for two prespecified contrasts, P90 versus P50 and P95 versus P50, for each exposure. Estimates were obtained from sensitivity analyses varying the degrees of freedom used for long-term temporal trend control (6, 7, and 8 per year) and the maximum lag period (5, 7, and 10 days). RR, relative risk; CI, confidence interval.

Supplementary Table S2. Distribution of candidate meteorological exposures during the study period.

Exposure	n	Mean	SD	Median	IQR	Min	Max	P10	P25	P50	P75	P90	P95	Zero count	Zero (%)
Mean temperature	4,383	17.357	9.118	18.054	15.681	-6.892	35.6	4.753	9.423	18.054	25.104	29.129	30.857	0	0.0
Maximum temperature	4,383	21.341	9.175	22.3	15.45	-5.4	40.7	8.4	13.4	22.3	28.85	33.1	34.7	1	0.02
Minimum temperature	4,383	13.868	9.437	14.4	16.2	-8.8	31.8	0.7	5.8	14.4	22.0	26.3	27.6	17	0.39
Relative humidity	4,383	74.979	11.877	75.708	16.542	33.083	99.458	58.417	67.125	75.708	83.667	90.417	93.042	0	0.0
Dew point	4,383	12.418	9.82	13.025	16.731	-19.717	27.804	-0.957	4.552	13.025	21.283	25.209	25.95	0	0.0
Absolute humidity	4,383	12.697	6.838	11.43	11.958	1.067	26.932	4.604	6.626	11.43	18.584	23.21	24.203	0	0.0
Precipitation	4,383	3.537	9.133	0.0	2.6	0.0	141.2	0.0	0.0	0.0	2.6	11.28	17.89	2257	51.49
Atmospheric pressure	4,383	1015.831	9.383	1015.796	15.529	985.367	1041.654	1003.768	1007.763	1015.796	1023.292	1028.436	1030.808	0	0.0
Mean wind speed	4,383	11.153	3.828	10.517	4.904	3.008	32.112	6.877	8.454	10.517	13.358	16.206	18.102	0	0.0
Maximum gust speed	4,383	32.513	9.148	31.0	11.9	13.0	86.4	22.0	25.9	31.0	37.8	44.6	49.7	0	0.0
Sunshine duration	4,383	7.708	4.374	9.632	6.838	0.0	14.0	0.0	4.236	9.632	11.074	12.131	12.766	462	10.54

Note: Values are summarized as mean, standard deviation, median, interquartile range, minimum, maximum, and selected percentiles. Zero counts and zero percentages are reported for variables with observed zero values. Units were °C for temperature and dew point, % for relative humidity, mm for precipitation, hPa for atmospheric pressure, km/h for wind speed and gust speed, hours for sunshine duration, and g/m³ for absolute humidity. SD, standard deviation; IQR, interquartile range; P10, P25, P50, P75, P90, and P95, 10th, 25th, 50th, 75th, 90th, and 95th percentiles, respectively.

Supplementary Table S3. Variable selection transparency for the distributed lag non-linear models.

Exposure	Prespecified priority	Rationale	Screening lag	Screening RR	Screening P value	Role in analysis	Interpretation
Mean temperature	Yes	Temperature-related metric with biological plausibility	Lag 0	1.394	<0.001	Comparative temperature metric, retained for completeness	Retained as a clinically relevant temperature metric; included for comparison because screening suggested signal but main DLNM stability was weaker
Relative humidity	Yes	Moisture-related metric with strong biological plausibility	Lag 3	1.107	<0.001	Primary moisture-related exposure	Retained because of biological plausibility, strong main-model signal, and stability across sensitivity analyses
Absolute humidity	Yes	Derived moisture-related metric reflecting absolute water content	Lag 3	1.353	<0.001	Supplementary comparative moisture metric	Retained as a related humidity-derived exposure for comparison; interpreted cautiously because precision was limited
Precipitation	Yes	Moisture-related environmental indicator with clinical interpretability	Lag 3	1.008	0.014	Primary main exposure (moisture-related environmental signal)	Retained because it captures an overlapping but clinically interpretable moisture-related environmental signal; interpreted as a shared signal rather than a formally independent effect

Note: This table summarizes the prespecified rationale, screening-stage signal, assigned analytic role, and interpretation for each exposure included in the main or comparative distributed lag non-linear models. Screening-stage estimates were used to inform model development and should not be interpreted as the primary effect estimates. RR, relative risk; DLNM, distributed lag non-linear model.

Supplementary Table S4. Diagnostics of the daily outpatient count series.

Observation days	Total visits	Mean daily count	Median daily count	Maximum daily count	Variance	Variance-to-mean ratio	Zero-visit days	Observed zero (%)	Expected zero under Poisson (%)
4,383	6,260	1.43	1	10	2.14	1.50	1,368	31.21	23.97

Note: The expected zero percentage under a Poisson distribution was calculated from the observed mean daily count. The variance-to-mean ratio was used to summarize overdispersion in the daily count series.

Supplementary Table S5. Diagnostic summary of the main quasi-Poisson distributed lag non-linear models.

Exposure	Model	Pearson dispersion	Ljung–Box P value, lag 7	Ljung–Box P value, lag 14	Ljung–Box P value, lag 21
Mean temperature	Quasi-Poisson DLNM	1.121	0.185	0.069	0.030
Relative humidity	Quasi-Poisson DLNM	1.121	0.167	0.051	0.026
Absolute humidity	Quasi-Poisson DLNM	1.123	0.190	0.049	0.022
Precipitation	Quasi-Poisson DLNM	1.124	0.148	0.094	0.036

Note: Pearson dispersion values and Ljung–Box test results are shown for the main quasi-Poisson distributed lag non-linear models. Residual autocorrelation function and partial autocorrelation function plots are presented in Supplementary Figure S6. The diagnostics were used to assess overdispersion and residual autocorrelation. DLNM, distributed lag non-linear model.

Supplementary Table S6. Lag-specific relative risks for the primary moisture-related exposures.

Exposure	Lag day	RR (95% CI), P95 vs P50
Relative humidity	0	0.970 (0.910–1.035)
Relative humidity	1	1.015 (0.977–1.055)
Relative humidity	2	1.054 (1.021–1.088)
Relative humidity	3	1.078 (1.037–1.120)
Relative humidity	4	1.078 (1.037–1.120)
Relative humidity	5	1.054 (1.021–1.089)
Relative humidity	6	1.015 (0.977–1.056)
Relative humidity	7	0.971 (0.910–1.035)
Precipitation	0	1.001 (0.966–1.037)
Precipitation	1	1.014 (0.992–1.036)
Precipitation	2	1.025 (1.006–1.043)
Precipitation	3	1.031 (1.009–1.053)
Precipitation	4	1.031 (1.009–1.053)
Precipitation	5	1.023 (1.005–1.042)
Precipitation	6	1.012 (0.990–1.034)
Precipitation	7	0.998 (0.964–1.034)

Note: Lag-specific relative risks are shown for the high-exposure contrast comparing the 95th percentile with the median level (P95 versus P50) across lag days 0–7. These estimates are lag-specific values rather than cumulative relative risks. RR, relative risk; CI, confidence interval.

Supplementary Table S7. Exploratory lag-block sensitivity analysis for overlapping moisture-related signals.

Exposure	Lag window	Model	RR (95% CI), P90 vs P50	RR (95% CI), P95 vs P50
Relative humidity	Lag days 2–5	Relative humidity block, main model	1.096 (1.051–1.143)	1.119 (1.063–1.178)
Relative humidity	Lag days 2–5	Relative humidity block, adjusted for rainy-day burden during lag days 2–5	1.071 (1.014–1.131)	1.088 (1.018–1.163)
Precipitation	Lag days 2–5	Precipitation block, main model	1.051 (1.017–1.086)	1.087 (1.028–1.149)
Precipitation	Lag days 2–5	Precipitation block, adjusted for relative humidity during lag days 2–5	1.004 (0.961–1.048)	1.006 (0.936–1.082)

Note: Estimates are shown for the prespecified lag-day window of 2–5 days. In the relative humidity analysis, the model was additionally adjusted for rainy-day burden within the same lag window. In the precipitation analysis, the model was additionally adjusted for relative humidity within the same lag window. These analyses were exploratory and were intended to assess whether relative humidity and precipitation reflected overlapping moisture-related environmental signals rather than mutually independent effects. Rainy-day burden was defined as the number of days with precipitation >0 mm during lag days 2–5. RR, relative risk; CI, confidence interval.
